# Elastic Constants and Related Properties of Ti_4_AC_3_ (A = Si, Au, Ir) MAX Phases: A Comparative First-Principles Study

**DOI:** 10.3390/ma19163452

**Published:** 2026-08-14

**Authors:** Guoqi Zhao, Yanlin Yu, Yufeng Wen

**Affiliations:** School of Science, Kaili University, Kaili 556011, China

**Keywords:** MAX phase, elastic property, mechanical property, thermal property, electronic structures, first principles

## Abstract

MAX-phase materials exhibit metallic–ceramic properties. The Ti_4_AC_3_ (A = Si, Au, Ir) 413-type MAX phases are investigated here using systematic density functional theory to examine structural stability, elastic behavior, mechanical performance, thermal properties, and electronic characteristics. Optimized lattice parameters agree with existing theoretical and experimental data. Single-crystal elastic constants satisfy the Born criteria, confirming mechanical stability. Using the Voigt–Reuss–Hill method, polycrystalline moduli are derived: Ti_4_IrC_3_ has the highest bulk modulus (*B* = 227.7 GPa), and Ti_4_SiC_3_ has the largest shear modulus (*G* = 148.1 GPa) and Young’s modulus (*E* = 354.5 GPa). Ti_4_AuC_3_ shows lower moduli, indicating greater ductility (*ν* = 0.293; *G*/*B* = 0.481). Elastic anisotropy is significant in all three phases, with Ti_4_AuC_3_ being the most anisotropic. Electronic density of states analysis reveals strong Ti–C covalent bonding that underpins stiffness, while A-site bonding (Si, Au, Ir) influences ductility and anisotropy. This comprehensive comparison elucidates the structure–property relationships of Ti_4_AC_3_ (A = Si, Au, Ir) MAX phases and supports their design for targeted applications in aerospace, energy, and mechanical engineering.

## 1. Introduction

MAX phases are a family of hexagonal layered ternary compounds with general chemical formulas M_2_AX or M_3_AX_2_ or M_4_AX_3_, where M is a transition metal element, A represents a *p*-block main-group element, and X corresponds to carbon or nitrogen [1]. These materials integrate the unique advantages of metals and ceramics, exhibiting outstanding comprehensive performance, including excellent high-temperature structural stability, superior machinability and ductility, high thermal conductivity, and prominent radiation resistance [2,3,4,5,6]. Benefiting from these excellent mechanical and thermal characteristics, MAX phases have emerged as promising candidate protective coating materials for key components serving in extreme harsh environments, such as advanced nuclear reactor systems and aeroengine thermal-end components [6]. Elastic stiffness constants are fundamental intrinsic physical parameters of crystalline materials. Accurate acquisition of elastic stiffness constants is a prerequisite for comprehensively and deeply understanding the mechanical behaviors and structural stability of crystals [7]. Furthermore, these elastic parameters are closely associated with multiple crucial thermal properties of crystals, including melting point, Debye temperature, thermal conductivity, and Grüneisen parameter, etc. Hence, systematic investigation of elastic stiffness constants is essential to reveal the intrinsic correlation between the microstructure and macroscopic mechanical/thermal performances of MAX phases.

First-principles calculations, one of the most reliable and effective theoretical approaches for predicting elastic stiffness constants, have been widely adopted to explore the physical properties of various MAX phases in recent decades. To date, extensive first-principles studies have focused on the elastic, mechanical, thermal, and electronic properties of M_2_AX phases, covering a large number of material systems, such as Sc_2_AC (A = Ga, In) [8], Nb_2_AC (A = Ga, Ge, Tl, Zn, P, In, and Cd) [9], (Y_2/3_M_1/3_)_2_AlX (M = Sc, Ti, Zr; X = C and N) [10], (V_2/3_Zr_1/3_)_2_AlC [11], M_2_CuB (M = Zr, Mo, and W) [12], Ti_2_BrX (X = B, C and N) [13], M_2_AlC (M = V, Nb, Ta) [14], M_2_GaC (M = V, Nb, Ta) [15,16], and Sc_2_AC (A = Al, Ga, In) [17]. Similarly, M_3_AX_2_ phases have also been extensively explored via first-principles calculations, with reported systems including M_3_AC_2_ (M = Zr, Hf; A = In, Pb, Cd, Sb) [17], M_3_PbC_2_ (M = Zr, Hf) [18], Zr_3_XC_2_ (X = Cd, Sb) [19], M_3_AC_2_ (M = Ti, V; A = Al, Si) [20], and M_3_AuC_2_ (M = Ti, V, and Cr) [21]. In contrast, existing theoretical research on M_4_AX_3_ phases remains relatively limited. Only a small number of M_4_AX_3_ systems have been systematically investigated, such as M_4_AN_3_ (M = Ti, Zr and Hf; A = Al and Si) [22], TM_4_AlN_3_ (TM = V, Nb, and Ta) [23], Ta_4_AlC_3_ [24], V_4_AlC_3_ [25], Ti_4_GeC_3_ [26], Ti_4_GaC_3_ [27], and Ti_4_SiC_3_ [3,28,29,30]. Notably, regarding Ti_4_AuC_3_ and Ti_4_IrC_3_ MAX phases, only their lattice parameters have been reported in the Supplementary Materials of a previous study [29]. No experimental or theoretical investigations have been conducted to explore their elastic, mechanical, thermal, and electronic properties, which leaves a significant research gap in the field. Against this background, this work adopts first-principles calculations to systematically investigate the elastic characteristics, mechanical performance, thermal behaviors, and electronic structures of Ti_4_AuC_3_ and Ti_4_IrC_3_. The classic Ti_4_SiC_3_ MAX phase is studied as a reference for comparative analysis. This study provides theoretical support for future engineering applications.

## 2. Methodology

**Figure 1:**
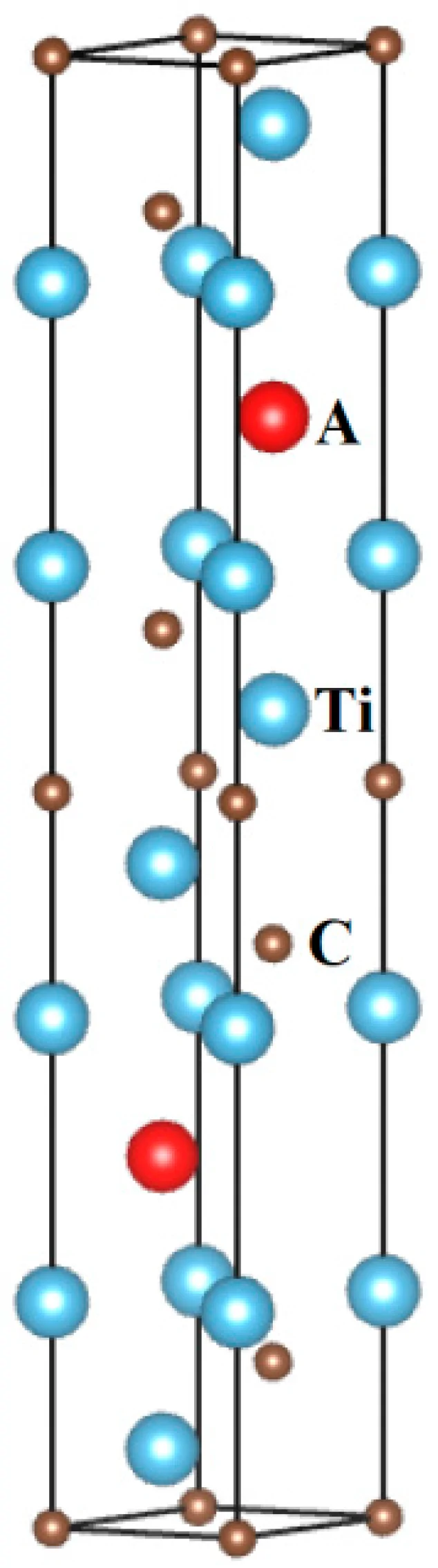
Crystal structure of Ti_4_AC_3_ (A = Si, Au and Ir) MAX phases.

The MAX phases Ti_4_SiC_3_, Ti_4_AuC_3_, and Ti_4_IrC_3_ crystallize usually in a hexagonal structure with the space group P6_3_/mmc [29]. Ti and Si/Au/Ir atoms are stacked in the order of ABABACBCBC along the c axis, as shown in Figure 1. An effective method to determine elastic stiffness constants, the stress–strain method [12] was used in this study to obtain the five independent single-crystal elastic stiffness constants: *C*_11_, *C*_12_, *C*_13_, *C*_33_, and *C*_44_ of these MAX phases. After performing first-principles calculations with full optimization of the lattice parameters and atomic coordinates of atoms in the unit cell, the elastic stiffness constants of these MAX phases were predicted using the strain-stress method based on first-principles calculations.

First-principles calculations were conducted based on density functional theory (DFT) using the projector augmented wave method [31] and Perdew–Burke–Ernzerhof (PBE) generalized gradient approximations (GGAs) [32], as implemented in the Vienna ab initio simulation package (VASP) [33,34,35]. An energy cutoff of 520 eV was applied for the expansion of electronic wave functions. A convergence criterion of 10^−6^ eV was applied to the total energy of the electronic self-consistency. The Brillouin zone was sampled using Γ-centered Monkhorst–Pack *k*-point grids [36]. The *k*-point mesh convergence test was conducted using grids ranging from 15 × 15 × 1 to 15 × 15 × 4. The total energy change per atom remained less than 2 meV/atom for 15 × 15 × 1 mesh and below 1 meV/atom for other tested meshes. A *k*-point grid of 15 × 15 × 1 was used for structural optimization and 21 × 21 × 2 for calculating elastic stiffness constants (*C_ij_*) and density of states (DOS). The structure was fully relaxed with respect to the volume, shape, and internal atomic positions until the atomic forces converged to less than 0.001 eV/Å per atom. The elastic tensor was determined by performing six finite distortions of the lattice and deriving the elastic constants from the strain–stress relationship using VASP. The elastic tensor was computed for both rigid and relaxed ions. Ionic contributions were determined by inverting the ionic Hessian matrix and multiplying it by the internal strain tensor [37,38]. The final elastic constants incorporated contributions from both rigid-ion distortions and ionic relaxations.

## 3. Results and Discussion

### 3.1. Elastic and Mechanical Properties

To investigate the mechanical properties of Ti_4_SiC_3_, Ti_4_AuC_3_, and Ti_4_IrC_3_ MAX phases, the elastic constants and elastic moduli were determined based on their optimized unit cells. Elastic constants represent the mechanical stability of the crystal structure, while elastic moduli quantify the material’s resistance to elastic deformation, including bulk modulus (*B*), shear modulus (*G*), and Young’s modulus (*E*). The optimized lattice parameters (*a*, *c*), volumes (*V*), and mass densities (*ρ*) of Ti_4_SiC_3_, Ti_4_AuC_3_, and Ti_4_IrC_3_ are listed in Table 1. For Ti_4_SiC_3_, the calculated lattice constants (*a* = 3.078 Å and *c* = 22.649 Å) are consistent with the previous theoretical results (3.08 Å and 22.62 Å) and the experimental values (3.05 Å and 22.67 Å) [3,28]. The optimized structural parameters (*a* = 3.078 Å, *c* = 22.649 Å, and *V* = 185.86 Å^3^) of Ti_4_SiC_3_ are also in excellent agreement with previous theoretical results (3.081 Å, 22.659 Å, and 186.26 Å^3^ [29] and 3.077 Å, 22.651 Å, and 185.8 Å^3^ [30]). The optimized structural parameters of Ti_4_AuC_3_ (3.085 Å, 23.590 Å, and 194.37 Å^3^) and Ti_4_IrC_3_ (3.045 Å, 23.052 Å, and 185.05 Å^3^) are in excellent agreement with previous theoretical results (3.087 Å, 23.584 Å, and 194.65 Å^3^ and 3.016 Å, 23.523 Å, and 185.27 Å^3^ [29], respectively).

The calculated elastic constants of Ti_4_SiC_3_, Ti_4_AuC_3_, and Ti_4_IrC_3_ are listed in Table 2. For Ti_4_SiC_3_, the calculated values (387.7, 93.8, 106.4, 363.3, 159.2 and 147.0 GPa) of the elastic constants *C*_11_, *C*_12_, *C*_13_, *C*_33_, *C*_44_, and *C*_66_ are in reasonable agreement with previous theoretical results (401.6, 84.6, 103.3, 384.3, 176.4 and 158.5 GPa [3] and 397.7, 172.5, 96.3, 378.4, 172.5 and 112.6 GPa [30]). Elastic constants can be used to assess mechanical stability. As proposed by Born and Huang [39], the mechanical stability of the hexagonal crystal system requires the following relationships to hold:

(1) $$\begin{array}{c} C_{ii}>0,i=1,3,4,6;\\ C^{\prime }=C_{11}-C_{12}>0;\\ C=\left(C_{11}+C_{12}\right)C_{33}-2C_{13}^{2}>0. \end{array}$$

Here, the constants *C_ii_* are diagonal entries of elastic stiffness matrix. The auxiliary stability parameter *C*′ ensures stability against shear deformation within the hexagonal basal plane. The auxiliary combined stability parameter *C* couples basal-plane stiffness and axial stiffness.

For the three MAX phases, *C*_11_ > 0, *C*_33_ > 0, *C*_44_ > 0, and *C*_66_ > 0. For Ti_4_SiC_3_, *C*′ = 293.9 > 0 and *C* = 152,246.2 > 0. Its elastic constants satisfy the conditions defined by Equation (1), and the results strongly demonstrate that Ti_4_SiC_3_ is mechanically stable. Similarly, for Ti_4_AuC_3_, *C*′ = 223.2 > 0, and *C* = 143,740.2 > 0. For Ti_4_IrC_3_, *C*′ = 298.3 > 0 and *C* = 180,466.6 > 0. The above data indicate that Ti_4_AuC_3_ and Ti_4_IrC_3_ exhibit mechanical stability. As suggested by Pettifor [40], Cauchy pressure (CP) can be used to classify the chemical bonding of solids. A positive CP indicates dominance of metallic bonding and a negative CP indicates dominance of angular bonding. The CP for hexagonal system is defined as: CP_1_ = *C*_13_–*C*_44_ and CP_2_ = *C*_12_–*C*_66_. The calculated values of CP_1_ and CP_2_ are listed in Table 2. For Ti_4_SiC_3_, the negative values of both CPs are in reasonable agreement with those of prior work [3]. The negative CPs indicate that the angular bonding is dominant in Ti_4_SiC_3_. Similarly, the metallic bonding is dominant in Ti_4_AuC_3_ because it has both positive CPs. However, Ti_4_IrC_3_ has a mixed nature of both metallic and angular bonding due to the opposite signs of CP_1_ and CP_2_. The positive CP_1_ larger than the absolute value of the negative CP_2_ indicates that the metallic bonding is more dominant in Ti_4_IrC_3_ than the angular bonding.

The bulk modulus *B* quantifies a crystal’s ability to resist volume changes and reflects the material’s resistance to fracture. The shear modulus *G* quantifies a crystal’s ability to resist shape changes and indicates its capacity to withstand plastic deformation. Generally, a high shear modulus indicates strong directional bonding characteristics among atoms. From the single-crystal elastic constants, the polycrystalline bulk modulus *B* and shear modulus *G* of hexagonal system are obtained using a Voigt–Reuss–Hill averaging scheme [41,42,43], and are expressed as follows:

(2) $$\begin{array}{l} B=\frac{1}{2}\left(B_{V}+B_{R}\right),G=\frac{1}{2}\left(G_{V}+G_{R}\right),\\ B_{V}=\frac{1}{9}\left[2\left(C_{11}+C_{12}\right)+4C_{13}+C_{33}\right],B_{R}=\frac{C}{M},\\ G_{V}=\frac{1}{30}\left[M+12\left(C_{44}+C_{66}\right)\right],G_{R}=\frac{5CC_{44}C_{66}}{6B_{V}C_{44}C_{66}+2C\left(C_{44}+C_{66}\right)},\\ M=C_{11}+C_{12}+2C_{33}-4C_{13},C=\left(C_{11}+C_{12}\right)C_{33}-2C_{13}^{2}. \end{array}$$

Here, *B* and *G* are Hill-averaged bulk modulus and shear modulus, respectively. The Voigt bound bulk modulus *B*_V_ is the upper limit of polycrystalline bulk modulus, the Reuss bound bulk modulus *B*_R_ is the lower limit of the polycrystalline bulk modulus, *G*_V_ represents the Voigt bound shear modulus, and *G*_R_ represents the Reuss bound shear modulus. Both *C* and *M* are the combination of elastic stiffness constants.

The calculated bulk and shear moduli are listed in Table 3. Among the three MAX phases, Ti_4_IrC_3_ exhibits the strongest resistance to volume changes due to its highest *B*, followed by Ti_4_SiC_3_, and Ti_4_AuC_3_ exhibits the weakest resistance to volume changes due to its lowest *B*. Similarly, the atomic bonding in Ti_4_SiC_3_ is the strongest due to its highest *G*, followed by Ti_4_IrC_3_, and the atomic bonding in Ti_4_AuC_3_ is the weakest due to its lowest *G*. Based on the value of *G*/*B*, it can be determined whether the material is ductile or brittle, where *G*/*B* = 0.571 is the critical value that distinguishes ductile materials from brittle materials [44]. As shown in Table 3, a value of 0.761 for Ti_4_SiC_3_ exhibits its brittleness, while those less than 0.571 for both Ti_4_AuC_3_ and Ti_4_IrC_3_ exhibit their ductility. The smaller *G*/*B* of Ti_4_AuC_3_ indicates that it is more ductile than Ti_4_IrC_3_.

Young’s modulus *E* characterizes a material’s resistance to tension or compression within its elastic limit. It is one of the key mechanical properties in the longitudinal direction. The Young’s modulus serves as an indicator of a material’s ease of elastic deformation: a larger value indicates that greater stress is required to induce a specific elastic deformation. Poisson’s ratio *σ* is also a key material property describing the relationship between axial and lateral strain in solid materials. The *E* and *σ* of the polycrystal are related to the bulk modulus *B* and shear modulus *G* through the following relation:

(3) $$\begin{array}{cc} E=\frac{9BG}{3B+G}, & \sigma =\frac{3B-2G}{6B+2G} \end{array}.$$

From the perspective of interatomic forces, the magnitude of the *E* reflects the strength of atomic bonding. As shown in Table 3, the modulus *E* of Ti_4_SiC_3_ is the highest, indicating the strongest interatomic bonding. In contrast, Ti_4_AuC_3_ exhibits the lowest *E*, indicating the weakest interatomic bonding. Poisson’s ratio (*σ*) provides more information about the characteristics of bonding forces than any other elastic constant [45]. The typical value of *σ* is 0.1, 0.25, and 0.33 for covalent, ionic, and metallic materials, respectively [46]. The *σ* value of 0.196 for Ti_4_SiC_3_ indicates its covalent/ionic bonding characteristics, while the value of *σ* for both Ti_4_AuC_3_ and Ti_4_IrC_3_ lies between 0.25 and 0.33, indicating that they exhibit ionic/metallic bonding characteristics. Ti_4_AuC_3_ has a larger *σ* value than Ti_4_IrC_3_, and thus it possesses stronger metallic bonding, which agrees with the analysis of the above Cauchy pressures. Moreover, the obtained *B*, *G*, *E*, and *σ* for Ti_4_SiC_3_ are consistent with the available results [3,30].

The isotropy and anisotropy of materials are widely recognized for their significant applications in engineering and crystal physics. Elastic constants are employed to describe the elastic anisotropy of materials. The anisotropy of materials with a hexagonal crystal structure is commonly quantified through the following anisotropy indexes [47,48,49,50]:

(4) $$\begin{array}{l} \Delta P=\frac{C_{33}}{C_{11}},\;\Delta S_{1}=\frac{C_{11}+C_{33}-2C_{13}}{4C_{44}},\;\Delta S_{2}=\frac{2C_{44}}{C_{11}-C_{12}},\\ A_{B}=\frac{B_{V}-B_{R}}{B_{V}+B_{R}}\times 100\%,\;A_{G}=\frac{G_{V}-G_{R}}{G_{V}+G_{R}}\times 100\%,\\ A^{U}=\frac{B_{V}}{B_{R}}+5\frac{G_{V}}{G_{R}}-6,\;A^{L}=\sqrt{{\left[\ln\left(\frac{B_{V}}{B_{R}}\right)\right]}^{2}+5{\left[\ln\left(\frac{G_{V}}{G_{R}}\right)\right]}^{2}}. \end{array}$$

Here, *C_ij_* are elastic stiffness tensor constants. ∆*P* denotes the anisotropic condition of the compressional wave within the crystal. Similarly, ∆S_1_ and ∆S_2_ denote the anisotropic conditions of the shear waves. If their values are equal to unity, the crystal exhibits elastic isotropy. Otherwise, it would be anisotropic [47]. The deviation from unity reflects the degree of anisotropy. *A_B_* is the bulk modulus anisotropy percentage from Voigt–Reuss bounds, and *A_G_* is the shear modulus anisotropy percentage from Voigt–Reuss bounds. *B*_V_ is the Voigt bound bulk modulus, and *B*_R_ is the Reuss bound bulk modulus. *G*_V_ is the Voigt bound shear modulus, and *G*_R_ is the Reuss bound shear modulus. If *A_B_* and *A_G_* are equal to zero, the crystal is elastically isotropic polycrystal, and larger absolute values indicate stronger anisotropy [48]. The universal anisotropy index *A*^U^ is linearly scaled with Voigt/Reuss ratios. The log-Euclidean anisotropy index *A*^L^ uses natural logarithms to reduce sensitivity to extreme modulus ratios. *A*^U^ and *A*^L^ are equal to zero for perfectly isotropic materials, and increasing positive values correspond to increasing elastic anisotropy [49,50]. The calculated values of these anisotropy indexes are listed in Table 4. For the three MAX phases, the non-unity values of ∆*P*, ∆S_1_, and ∆S_2_ indicate that they are elastically anisotropic. For the compressional wave, Ti_4_SiC_3_ exhibits the strongest anisotropy, while both Ti_4_AuC_3_ and Ti_4_IrC_3_ are almost isotropic. For shear waves, Ti_4_AuC_3_ exhibits the strongest anisotropy, followed by Ti_4_IrC_3_, and Ti_4_SiC_3_ exhibits the weakest anisotropy. Similarly, the non-zero values of *A_B_*, *A_G_*, *A*^U^, and *A*^L^ also indicate that they are elastically anisotropic. For each of *A_B_*, *A_G_*, *A*^U^, and *A*^L^, Ti_4_AuC_3_ has the largest value, followed by Ti_4_IrC_3_, and Ti_4_SiC_3_ has the smallest value. These also indicate that Ti_4_AuC_3_ exhibits the strongest anisotropy, followed by Ti_4_IrC_3_, and Ti_4_SiC_3_ exhibits the weakest anisotropy.

In addition to anisotropy indexes, the directional dependence of elastic moduli can also characterize the elastic anisotropy of materials. For a hexagonal crystal, the directional dependence of Young’s modulus *E* is expressed as [51]:

(5) $$E^{-1}=S_{11}{\left(\alpha^{2}+\beta^{2}\right)}^{2}+S_{33}\gamma^{4}+\left(2S_{13}+S_{44}\right)\left(\beta^{2}\gamma^{2}+\alpha^{2}\gamma^{2}\right).$$

**Figure 2:**
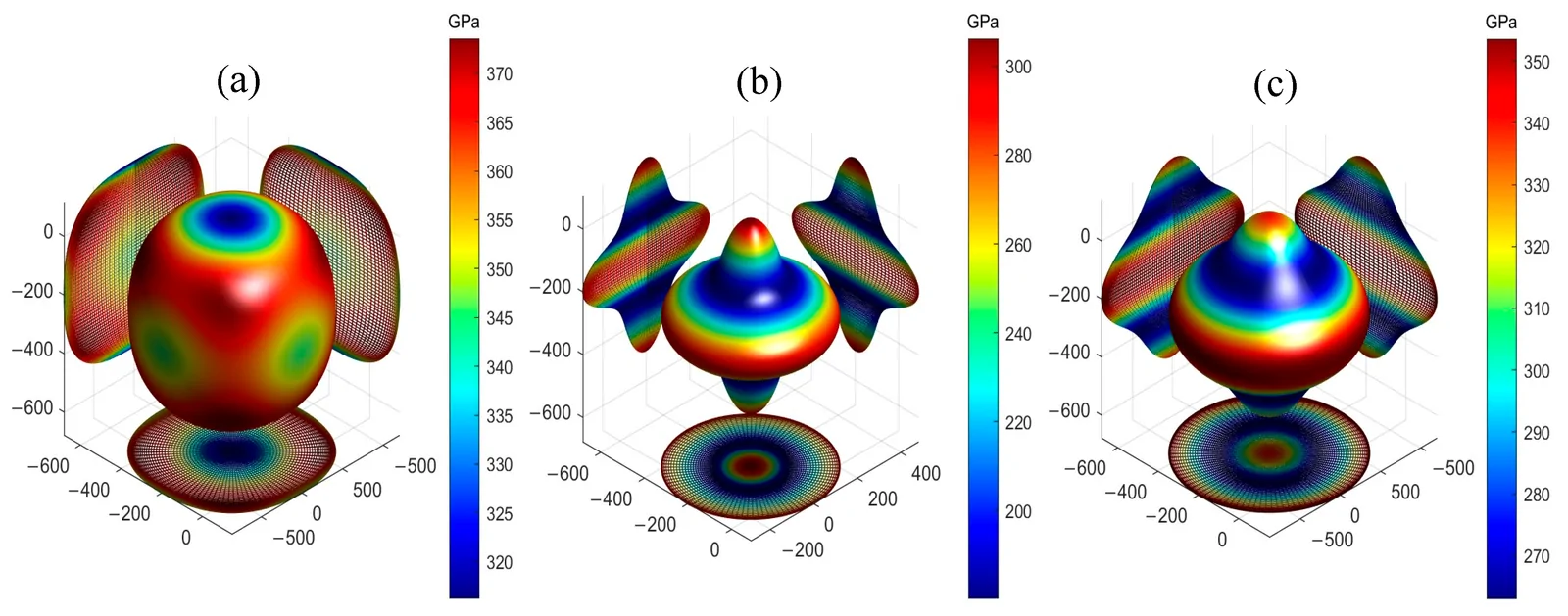
Directional dependence of Young’s modulus for MAX phases: (**a**) Ti_4_SiC_3_, (**b**) Ti_4_AuC_3,_ and (**c**) Ti_4_IrC_3_.

**Figure 3:**
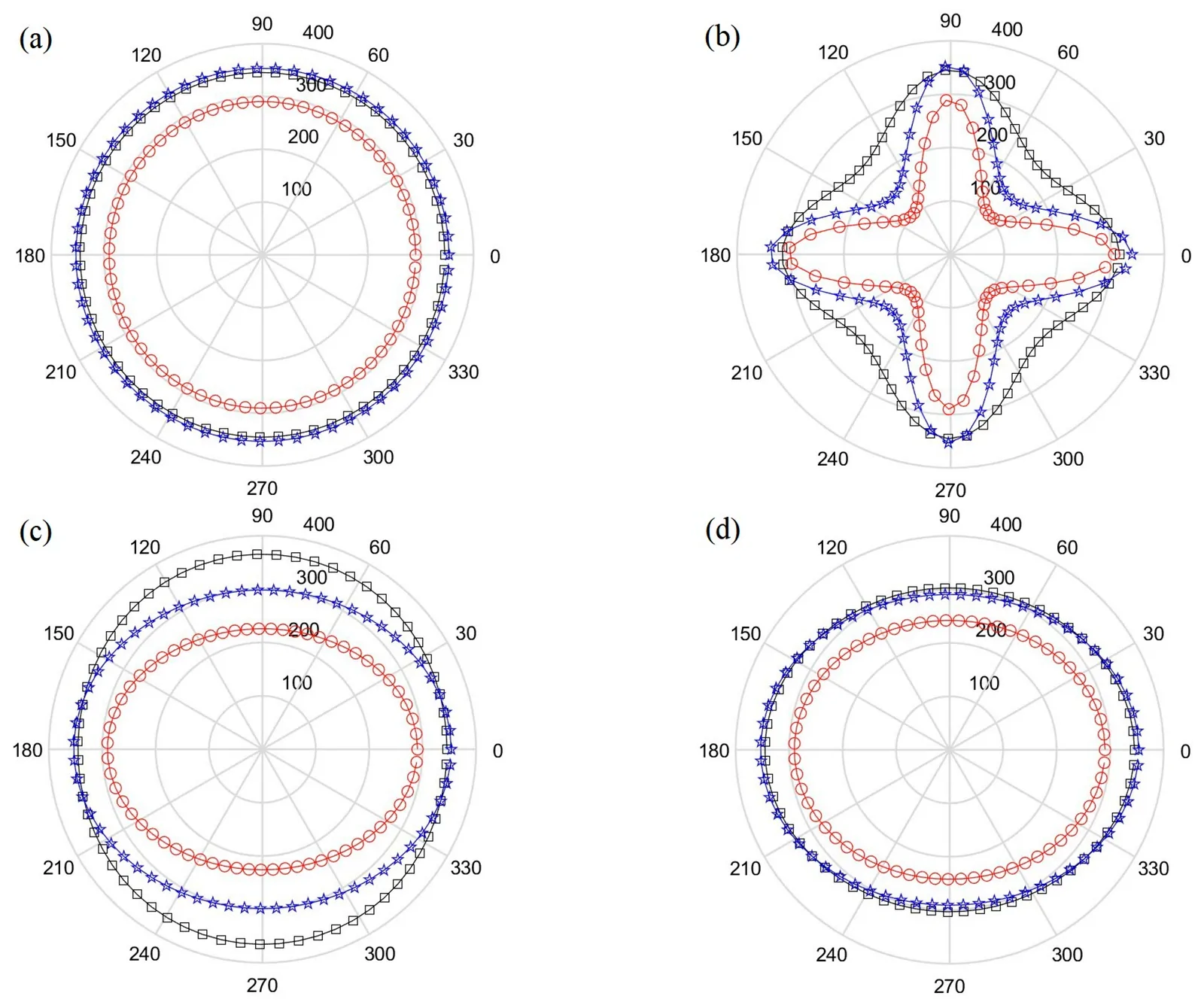
Projections in different planes of directionally dependent Young’s modulus for Ti_4_SiC_3_ (black square), Ti_4_AuC_3_ (red circle), and Ti_4_IrC_3_ (blue pentagram): (**a**) (0001) basal plane, (**b**) (10$\bar{1}$0) or ($\bar{1}$2$\bar{1}$0) prismatic plane, (**c**) ($\bar{1}$012) pyramidal plane, and (**d**) (1$\bar{2}$12) pyramidal plane.

Here, *S_ij_* are the elements of the elastic compliance tensor that satisfies [*S_ij_*] = [*C_ij_*]^−1^; *α* = *l*_11_, *β* = *l*_12_ and *γ* = *l*_13_ are the direction cosines. The calculated Young’s moduli along different directions are shown in Figure 2. A spherical shape in the directionally dependent Young’s modulus will be obtained for an isotropic crystal. The nonspherical nature of Young’s modulus for the three MAX phases clearly shows the anisotropy in their Young’s moduli. The deviation from sphericity reflects the degree of anisotropy. Obviously, Ti_4_AuC_3_ exhibits the strongest anisotropy because its deviation from sphericity is the greatest, followed by Ti_4_IrC_3_, and Ti_4_SiC_3_ exhibits the weakest anisotropy because its deviation from sphericity is the smallest. Furthermore, their projections of Young’s modulus on the different planes are shown in Figure 3. For the three MAX phases, Young’s modulus is orientation-independent on the basal plane, as shown in Figure 3a. However, it is strongly orientation-dependent on the prismatic and the pyramidal planes, as shown in Figure 3b–d. Similarly, the deviation from circular also reflects the degree of anisotropy. The elastic anisotropy on the prismatic plane (10$\bar{1}$0) or ($\bar{1}$2$\bar{1}$0) and the pyramidal planes ($\bar{1}$012) and (1$\bar{2}$12) for Ti_4_AuC_3_ is the strongest, followed by Ti_4_IrC_3_, and that for Ti_4_SiC_3_ is the weakest.

### 3.2. Thermal Properties

Debye temperature (*Θ*_D_), a key parameter for crystalline materials, is used to characterize the thermal properties of a material and their correlation with mechanical properties. It can be derived from the mean sound velocity (*υ*_M_) by the following relation [52]:

(6) $$\begin{array}{cc} \Theta_{D}=\frac{h}{k_{B}}{\left(\frac{3nN_{A}\rho }{4\pi M}\right)}^{1/3}\upsilon_{M}, & \upsilon_{M}=\sqrt[{3}]{3}{\left(\frac{1}{\upsilon_{L}^{3}}+\frac{2}{\upsilon_{T}^{3}}\right)}^{-1/3} \end{array}.$$

Here, *h* and *k*_B_ represent Plank’s and Boltzmann’s constants, respectively; *n* is the number of atoms per formula unit; *N*_A_ is Avogadro’s number; *ρ* is the mass density; *M* is the molecular mass per formula unit; and *υ*_L_ and *υ*_T_ are longitudinal and transverse elastic wave velocities, respectively, derived from Navier’s equations [53]:

(7) $$\begin{array}{cc} \upsilon_{L}={\left(\frac{3B+4G}{3\rho }\right)}^{1/2}, & \upsilon_{T}={\left(\frac{G}{\rho }\right)}^{1/2} \end{array}.$$

Here, *B* and *G* are Hill-averaged bulk modulus and shear modulus, respectively, and *ρ* is the mass density. Lattice thermal conductivity (*κ*_ph_) is a fundamental and critical physical property of MAX phases, making it highly relevant for high-temperature applications. The lattice thermal conductivity can be expressed using the Slack equation based on the Debye model [54] as:

(8) $$\kappa_{\mathrm{ph}}=A\frac{\bar{M}\Theta_{D}^{3}\delta }{\gamma^{2}n^{2/3}T}.$$

Here, *κ*_ph_ is lattice thermal conductivity, $\bar{M}$ refers to the average atomic mass (in kg/mol) in a molecule, *Θ*_D_ is Debye temperature, *δ* denotes the cubic root of average atomic volume, *n* refers to the number of atoms present in the unit cell, *T* is the Kelvin temperature, *γ* denotes the Grüneisen constant, and *A* is a coefficient (in (W·mol)/(kg·m^2^·K^3^)) depending on the constant *γ*. The acoustic *γ* is defined as [55]:

(9) $$\gamma =\frac{9\upsilon_{L}^{2}-12\upsilon_{T}^{2}}{2\upsilon_{L}^{2}+4\upsilon_{T}^{2}}=\frac{3\left(1+\sigma \right)}{2\left(2-3\sigma \right)}.$$

Here, *υ*_L_ and *υ*_T_ are longitudinal and transverse elastic wave velocities, respectively, and *σ* is Poisson’s ratio. Using the Julian formula [56], the coefficient *A* (*γ*) was determined as:

(10) $$A\left(\gamma \right)=\frac{5.720\times 10^{7}\times 0.849}{2\left(1-0.514/\gamma +0.228/\gamma^{2}\right)}.$$

The Slack model relies on several simplifying assumptions. Under the isotropic Debye approximation, it is valid only at high temperatures in which intrinsic three-phonon scattering dominates. It describes ideal defect-free crystals and ignores extrinsic scattering. It treats the Grüneisen parameter as a constant average and omits higher-order phonon interactions. Because it lacks mode-resolved phonon information, the semi-empirical formula enables rapid screening, but provides only semiquantitative predictions rather than rigorous quantitative results.

At high temperatures, phonons decouple, and thermal energy is transferred between adjacent atoms. In this scenario, the lattice thermal conductivity converges to a minimum value (*κ*_min_) independently of temperature [57]. Under this constraint, the phonon mean free path can be approximated by the average interatomic distance. Consequently, the atoms in a molecule are replaced with equivalent atoms, where the mass of an equivalent atom is the average atomic mass *M*/*n*. The minimum lattice thermal conductivity can be determined using the following equation [58]:

(11) $$\kappa_{\min}=k_{B}\upsilon_{M}{\left(\frac{M}{n\rho N_{A}}\right)}^{-2/3}.$$

Here, *κ*_min_ represents minimum lattice thermal conductivity; *υ*_M_ denotes the mean sound velocity; *M* is the molecular mass per formula unit; *n* is the number of atoms per formula unit; *ρ* is the mass density; and *N*_A_ is Avogadro’s number. This model establishes a universal theoretical lower bound for thermal conductivity under strong scattering conditions, but yields only qualitative reference values. It does not provide rigorous quantitative predictions, particularly for layered, low-symmetry crystalline systems. The melting temperature (*T*_m_) of hexagonal crystals can be calculated using the elastic constants *C_ij_* [59] as:

(12) $$T_{m}=354+1.5\frac{\left(2C_{11}+C_{33}\right)}{\mathrm{GPa}}K.$$

Here, *C*_11_ is longitudinal stiffness within hexagonal basal plane, and *C*_33_ is longitudinal stiffness along hexagonal *c*-axis. This empirical relation for melting temperature *T*_m_ is derived from linear regression and uses only two elastic constants. If thermal modulus softening, defects, and thermodynamic free energy are neglected, the fitted coefficients are specific to material families. This provides rapid qualitative estimates, but cannot yield universal, quantitatively reliable predictions.

**Figure 4:**
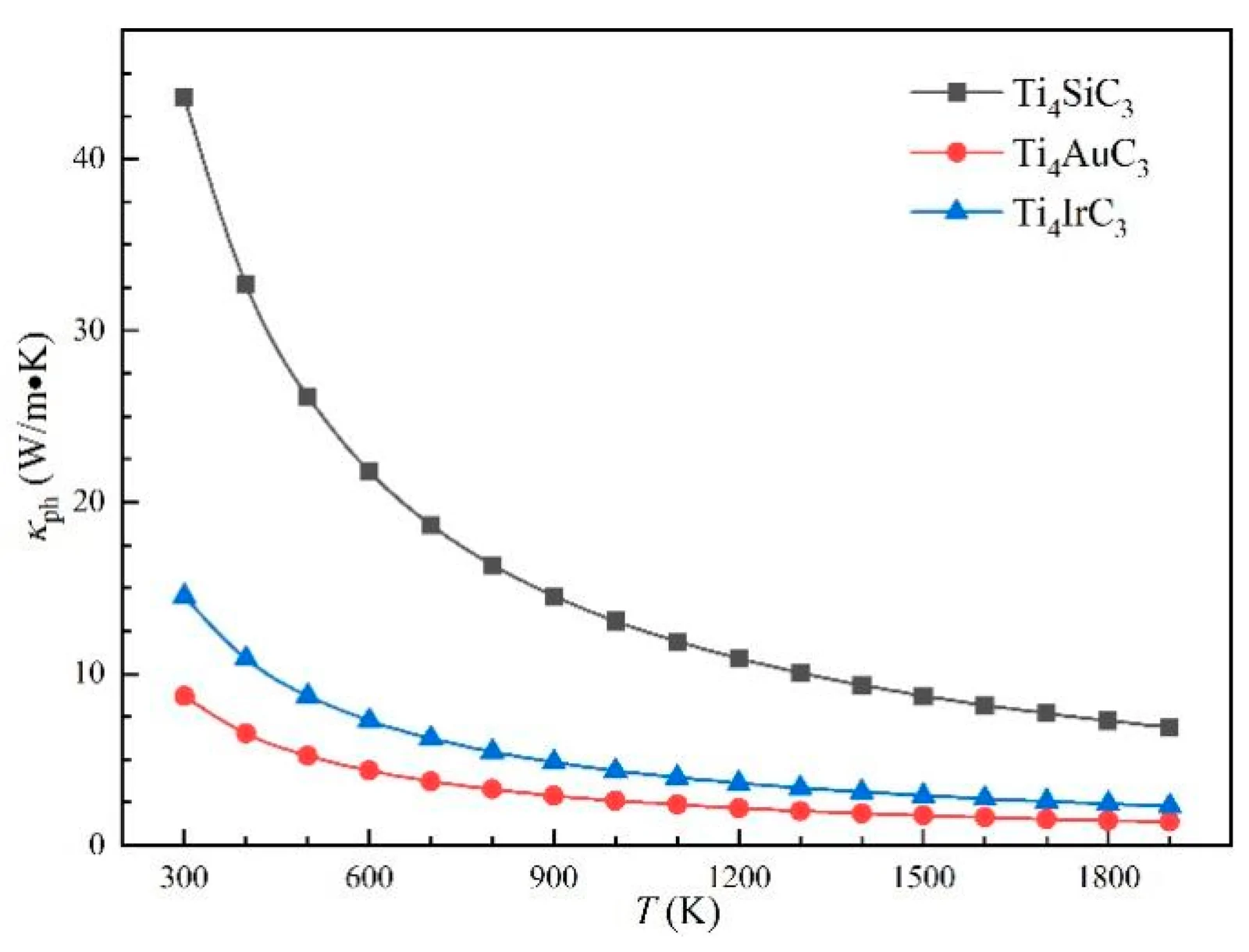
Lattice thermal conductivity (*κ*_ph_) of Ti_4_SiC_3_, Ti_4_AuC_3_, and Ti_4_IrC_3_ MAX phases as a function of temperature (*T*).

The calculated sound velocity, Debye temperature, Grüneisen constant, lattice thermal conductivity at 300 K, minimum thermal conductivity, and melting temperature are listed in Table 5. The results of *υ*_L_ (9264 m/s), *υ*_T_ (5694 m/s), and *Θ*_D_ (825.9 K) for Ti_4_SiC_3_ are basically consistent with the previously theoretical values of 9490 m/s, 5940 m/s, and 860.4 K, respectively. The value 825.9 K of *Θ*_D_ for Ti_4_SiC_3_ is in good agreement with the available theoretical datum of 849.7 K [30]. Among the three MAX phases, Ti_4_SiC_3_ possesses the highest *Θ*_D_, followed by Ti_4_IrC_3_, and Ti_4_AuC_3_ possesses the lowest *Θ*_D_. At the atomic level, the Debye temperature reflects the strength of interatomic bonding, with a higher Debye temperature indicating stronger interatomic bonds [60]. Therefore, the interatomic bonding strength in Ti_4_SiC_3_ is the strongest, followed by Ti_4_IrC_3_, and that in Ti_4_AuC_3_ is the weakest. Similarly, higher Debye temperature of the materials corresponds to their higher thermal conductivity. Thus, Ti_4_SiC_3_ has the highest thermal conductivity, followed by Ti_4_IrC_3_, and Ti_4_AuC_3_ has the lowest thermal conductivity. For the three MAX phases, Figure 4 shows that the lattice thermal conductivity decreases gradually with increasing temperature. In the studied entire temperature range, the lattice thermal conductivity of Ti_4_SiC_3_ is the highest, followed by Ti_4_IrC_3_, and that of Ti_4_AuC_3_ is the lowest. The minimum thermal conductivity is directly proportional to the mean sound velocity in crystalline solids. The *κ*_min_ of Ti_4_SiC_3_ is the largest owing to its largest *υ*_M_, followed by Ti_4_IrC_3_, and that of Ti_4_AuC_3_ is the smallest owing to its smallest *υ*_M_. However, the *T*_m_ of Ti_4_IrC_3_ is the highest due to its largest *C*_11_ and *C*_33_ (seen in Table 2), followed by Ti_4_SiC_3_, and that of Ti_4_AuC_3_ is the lowest due to its smallest *C*_11_ and *C*_33_. To the best of our knowledge, no experimental and theoretical data are available for meaningful comparison. Therefore, the present results serve as a prediction for future experimental studies.

### 3.3. Electronic Structures

**Figure 5:**
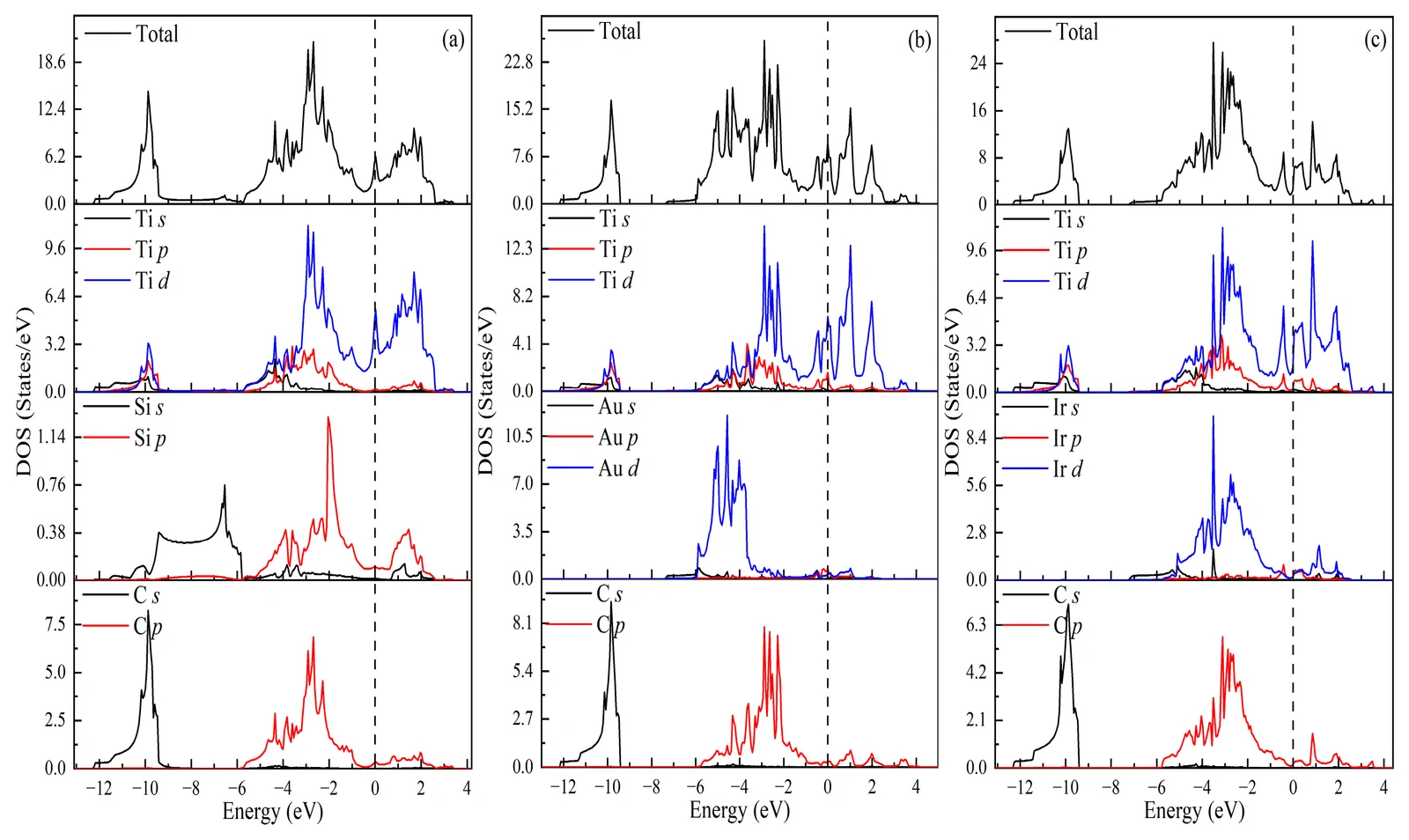
Density of states (DOS) for (**a**) Ti_4_SiC_3_, (**b**) Ti_4_AuC_3_, and (**c**) Ti_4_IrC_3_ MAX phases. The Fermi level is shifted to 0 eV.

The DOS describes the distribution of electrons among available orbitals and the interactions between atoms, and it reveals the nature of chemical bonding in a compound [61]. Figure 5a–c present the total DOS and the partial DOS for Ti 3p, 3d and 4s; C 2s and 2p; Si 3s and 3p; Au 5p, 5d and 6s; and Ir 5p, 5d and 6s in Ti_4_SiC_3_, Ti_4_AuC_3_ and Ti_4_IrC_3_. The total DOS at the Fermi level (E_F_) for each phase is peaked, reflecting a large DOS and strong metallicity. In the region below approximately −9.4 eV, the total DOS is dominated by C 2s and Ti 3d, 3p, 4s states, with a strong peak near −10 eV arising mainly from C 2s and Ti 3d contributions. In the region from about −6 eV and 4 eV, Ti 3d states contribute significantly to the total DOS and show a pronounced peak near the E_F_, indicating that Ti.

3d orbitals strongly influence the electronic structure. Ti 3p states also contribute modestly in this energy range. Ti 4s states contribute only modestly to the DOS between roughly −6 eV and −2 eV. C 2p states contribute significantly to the electronic states between approximately −6 eV and around 2 eV, with a peak near the E_F_ indicating a substantial DOS contribution. For Ti_4_SiC_3_, the Si 3s orbital contributes negligibly, while the Si 3p orbital contributes substantially in the energy range from about −6 eV to around 2 eV, as shown in Figure 5a. Overlaps occur among Ti 3p, Ti 3d, Si 3p and C 2p states near the E_F_. For Ti_4_AuC_3_, the Au 5d orbital is the dominant contributor to the DOS in the energy range from about −6 eV to −3.4 eV, as shown in Figure 5b. There are overlaps among Ti 3p, Ti 3d, Au 5d and C 2p states near the E_F_. For Ti_4_IrC_3_, Ir 5d orbital contributes significantly to the electronic states in the energy range from about −6 eV to 2 eV, with a pronounced peak near the E_F_, indicating their substantial contribution to the total DOS (see in Figure 5c). Overlaps persist among Ti 3p, Ti 3d, Ir 5d, and C 2p states near the E_F_.

**Figure 6:**
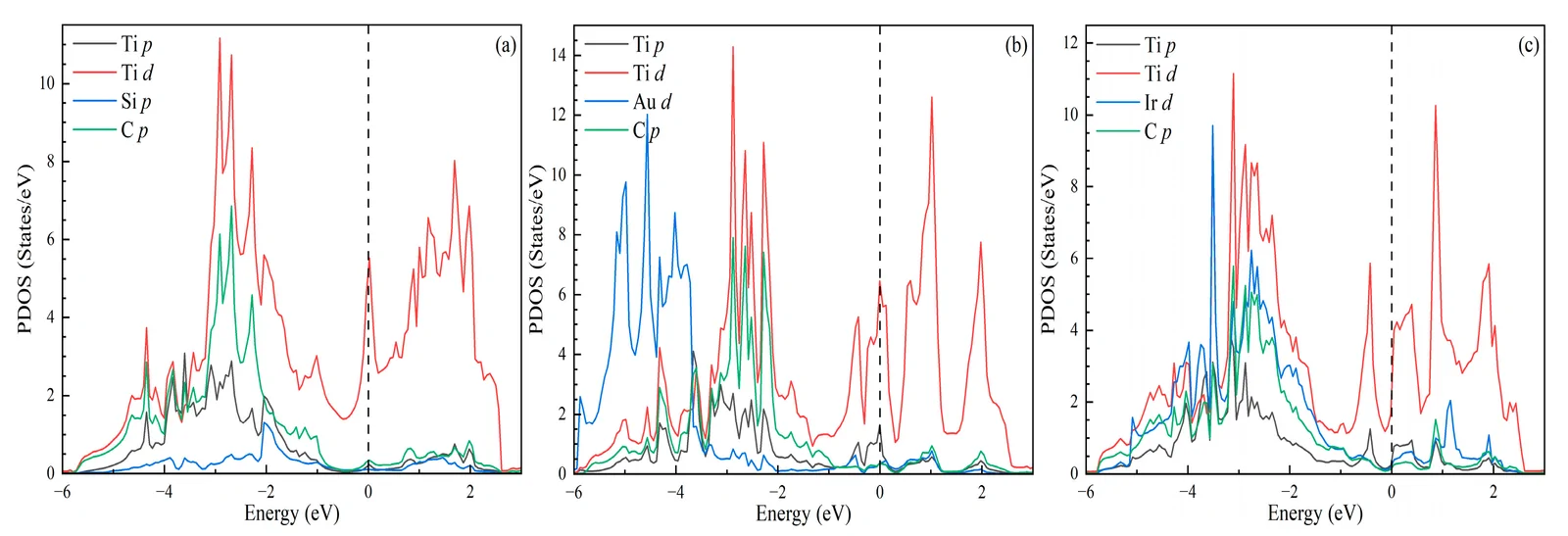
Partial density of states (PDOS) for (**a**) Ti_4_SiC_3_, (**b**) Ti_4_AuC_3_, and (**c**) Ti_4_IrC_3_ MAX phases. The Fermi level is shifted to 0 eV.

Overlaps between distinct orbitals signal potential orbital hybridization and the emergence of complex chemical bonding. Figure 6a–c illustrate the partial DOS near the *E*_F_ for Ti 3*p* and 3*d*, C 2*p*, Si 3*p*, Au 5*d*, and Ir 5*d* orbitals in Ti_4_SiC_3_, Ti_4_AuC_3_ and Ti_4_IrC_3_. For all three phases, continuous Ti 3*d* states reveal the delocalization of transition-metal *d* electrons, confirming metallic bonding character. Prominent peak overlaps between Ti 3*p*/3*d* and the C 2*p* states in each phase demonstrates strong *p*–*p* and *p*–*d* hybridization between Ti and C, indicative of robust covalent Ti–C bonds. In Ti_4_SiC_3_, noticeable overlap occurs between the Ti 3*p*/3*d* and Si 3*p* states (Figure 6a), confirming Ti–Si *p*–*p* and *p*–*d* hybridization and covalent Ti–Si bonding. Overlapping C 2*p* and Si 3*p* peaks further reveal C–Si *p*–*p* hybridization, supporting covalent C–Si interactions. For Ti_4_AuC_3_, partial overlap Ti 3*d*/3*p* and Au 5*d* states (Figure 6b) imply *d*–*d* and *p*–*d* hybridization, forming Ti–Au metallic bonds. The weak orbital overlap between C 2*p* and Au 5*d* suggests limited covalent interaction between C and Au. In Ti_4_IrC_3_, strong interaction between Ir 5*d* and Ti 3*d*/3*p* orbitals (Figure 6c) produces metallic Ir–Ti bonds. Significant overlap between C 2*p* and Ir 5*d* orbitals demonstrates strong covalent C–Ir bonding. These electronic structure findings align well with the preceding Cauchy pressure analysis. Such electronic structure insights establish a mechanistic link between partial DOS characteristics and the divergent mechanical behavior observed across the three MAX phases.

## 4. Conclusions

This study systematically investigated the structural, mechanical, elastic, thermal, and electronic properties of Ti_4_SiC_3_, Ti_4_SiC_3_, and Ti_4_SiC_3_ MAX phases via first-principles DFT calculations. The optimized lattice parameters are consistent with available theoretical and experimental results, and the single-crystal elastic constants confirm that all three Ti_4_AC_3_ structures are mechanically stable. Key mechanical descriptors, including Cauchy pressures, polycrystalline elastic moduli, directional stiffness, and elastic anisotropy, were comprehensively evaluated to distinguish their intrinsic mechanical behaviors. The mechanical results reveal prominent differences in ductility and anisotropy across the three phases. Based on the Cauchy pressure, *G*/*B* ratio, and Poisson’s ratio, the ductility decreases in the order Ti_4_AuC_3_ > Ti_4_IrC_3_ > Ti_4_SiC_3_. The axial incompressibility follows the trend Ti_4_IrC_3_ > Ti_4_SiC_3_ > Ti_4_AuC_3_, while Ti_4_IrC_3_ and Ti_4_AuC_3_ exhibit higher elastic anisotropy than Ti_4_SiC_3_. Elastic modulus-derived thermal properties exhibit systematic variations among the studied MAX phases. Ti_4_SiC_3_ shows the highest sound velocities, Debye temperature, and thermal conductivity, followed by Ti_4_IrC_3_ and Ti_4_AuC_3_. Conversely, the Grüneisen parameter decreases from Ti_4_AuC_3_ to Ti_4_IrC_3_ and Ti_4_SiC_3_, whereas the melting point increases sequentially for Ti_4_AuC_3_, Ti_4_SiC_3_, and Ti_4_IrC_3_. These findings demonstrate clear trade-offs among ductility, structural stiffness, elastic anisotropy, and thermal transport performance in Ti_4_AC_3_ systems. The property distinctions indicate that Ti_4_AuC_3_ is favorable for ductile and deformable coating applications, while high-stiffness and -melting-point Ti_4_IrC_3_ is suitable for high-temperature extreme environments and thermally conductive Ti_4_ASiC_3_ serves well for heat-dissipation components. This work provides reliable theoretical guidelines for the future design and performance optimization of novel Ti_4_AC_3_ MAX phases. Further studies involving experimental verification, finite-temperature simulations, and phonon-based thermal transport analyses are recommended to advance the practical engineering application of these materials.

## Figures and Tables

**Table 1:** Optimized lattice parameters (*a*, *c* in Å), volume per unit cell (*V* in Å^3^), and mass density (*ρ*, in g/cm^3^) of Ti_4_AC_3_ (A = Si, Ir and Au) phases, with available theoretical and experimental values [3,28,29].

Phase	*a*	*c*	*V*	*ρ*	
Ti_4_SiC_3_	3.078	22.649	185.86	4.569	This
Theo.	3.08	22.62			[3,28]
	3.081	22.659	186.26		[29]
	3.077	22.651	185.8		[30]
Expt.	3.05	22.67			[3,28]
Ti_4_AuC_3_	3.085	23.590	194.37	7.255	This
	3.087	23.584	194.65		[29]
Ti_4_IrC_3_	3.045	23.052	185.05	7.536	This
	3.016	23.523	185.27		[29]

**Table 2:** Elastic constants (*C_ij_*, in GPa) of Ti_4_AC_3_ (A = Si, Ir and Au) phases, with available data [3,30].

Phase	*C*_11_	*C*_12_	*C*_13_	*C*_33_	*C*_44_	*C*_66_	CP_1_	CP_2_	
Ti_4_SiC_3_	387.7	93.8	106.4	363.3	159.2	147.0	−52.8	−53.2	This
Theo.	401.6	84.6	103.3	384.3	176.4	158.5	−73.1	−73.9	[3]
	397.7	172.5	96.3	378.4	172.5	112.6			[30]
Ti_4_AuC_3_	346.5	123.3	96.5	345.7	59.4	111.6	37.1	11.7	This
Ti_4_IrC_3_	414.9	116.6	142.7	416.1	90.8	149.2	51.9	−32.6	This

**Table 3:** Bulk, shear and Young’s moduli (GPa) and Poisson’s ratio of Ti_4_AC_3_ (A = Si, Ir and Au) phases.

Phase	*B*_V_	*G*_V_	*B*_R_	*G*_R_	*B*	*G*	*G*/*B*	*E*	*σ*	Ref.
Ti_4_SiC_3_	194.7	148.5	194.6	147.7	194.6	148.1	0.761	354.5	0.196	This
					196.7	161.4	0.82	380.3	0.18	[3]
					196.6	157.1	0.80			[30]
Ti_4_AuC_3_	185.7	94.2	185.5	84.3	185.6	89.3	0.481	230.8	0.293	This
Ti_4_IrC_3_	227.8	122.4	227.6	116.3	227.7	119.3	0.524	304.8	0.277	This

**Table 4:** Anisotropy factors of Ti_4_AC_3_ (A = Si, Ir and Au) phases.

Phase	Δ*P*	Δ*S*_1_	Δ*S*_2_	*A_B_*	*A_G_*	*A* ^U^	*A* ^L^
Ti_4_SiC_3_	0.937	0.845	1.083	0.01	0.27	0.027	0.012
Ti_4_AuC_3_	0.998	2.099	0.533	0.06	5.58	0.592	0.250
Ti_4_IrC_3_	1.003	1.502	0.609	0.05	2.58	0.266	0.115

**Table 5:** Longitudinal (*υ*_L_), transverse (*υ*_T_), and mean (*υ*_M_) sound velocities (m/s), Debye temperature (*Θ*_D_, in K), Grüneisen constant (*γ*), lattice thermal conductivity (*κ*_ph_, in W/(m K)) at 300 K, minimum thermal conductivity (*κ*_min_, in W/(m K)), and melting point (*T*_m_, in K) of Ti_4_AC_3_ (A = Si, Ir and Au) phases.

Phase	*υ*_L_	*υ*_T_	*υ*_M_	*Θ*_D_	*γ*	*κ*_ph_	*κ*_min_	*T*_m_	Ref.
Ti_4_SiC_3_	9264	5694	6284	825.9	1.272	43.57	1.692	2062	This
	9490	5940		860.4					[3]
				849.7					[30]
Ti_4_AuC_3_	6479	3507	3914	506.8	1.729	8.69	1.023	1912	This
Ti_4_IrC_3_	7164	3980	4432	583.4	1.638	14.52	1.197	2223	This

## Data Availability

The original contributions presented in this study are included in the article. Further inquiries can be directed to the corresponding author.
